# Intracranial bleeding in acute promyelocytic leukemia treated with arsenic trioxide based regimens is associated with induction mortality but not with relapse

**DOI:** 10.1038/s41408-023-00873-z

**Published:** 2023-06-22

**Authors:** Uday Prakash Kulkarni, Sushil Selvarajan, N. A. Fouzia, Sharon Lionel, Sukesh Chandran Nair, Poonkuzhali Balasubramanian, Thenmozhi Mani, Aby Abraham, Biju George, Vikram Mathews

**Affiliations:** 1 Department of Haematology, Christian Medical College, Vellore, India; 2 Department of Transfusion Medicine and Immunohaematology, Christian Medical College, Vellore, India; 3 Department of Biostatistics, Christian Medical College, Vellore, India

**Keywords:** Acute myeloid leukaemia, Risk factors

## To The Editor:

**Fig. 1:**
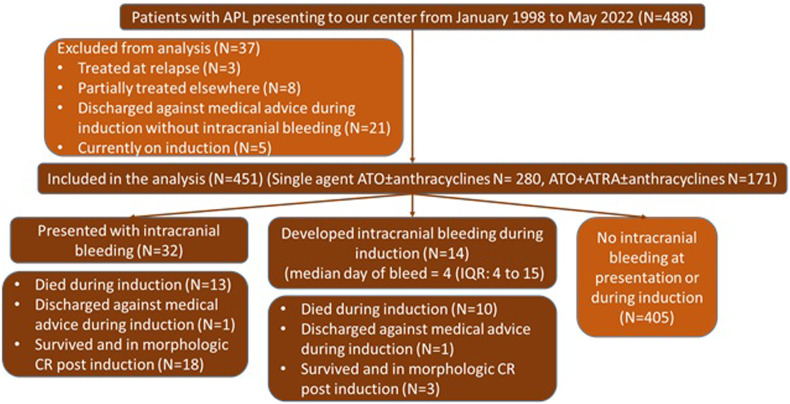
Study flowchart for the current retrospective analysis.

Intracranial bleeding (ICB), either at presentation or during induction, is known to be associated with early deaths in acute promyelocytic leukemia (APL). With ATRA and chemotherapy treatment, high WBC count at presentation and prior CNS hemorrhage are risk factors for a subsequent CNS relapse (HR – 9.9, 95% CI 3.32–57.40; *P*-value 0.006) [1]. However, it is not known if this remains true with the advent of upfront arsenic trioxide (ATO) based regimens. At our center, we have been using an upfront ATO-based treatment regimen for APL for more than 2 decades. From the year 1998 to 2015, single agent ATO was used [2] while after 2015, a combination of all-trans retinoic acid and ATO with minimal anthracyclines [3]. None of the patients received any CNS-directed therapy. During induction, all patients received transfusion support to target a hemoglobin of 8 g%, platelet count of 30 × 10^9^/L, normal prothrombin time, normal activated partial thromboplastin time, and fibrinogen above 150 mg% for the first 2 weeks. In the presence of ICB, the platelet target was 50 × 10^9^/L, the coagulation parameters were measured at least twice daily, and the coagulation support (irradiated packed cells, irradiated platelet-rich concentrates, fresh frozen plasma, and cryoprecipitates) was continued for at least 2 weeks following the bleeding. After institutional ethics committee approval (IRB: 11380 (OBSERVE) dated: 27.06.2018), we conducted a retrospective analysis of all patients with newly diagnosed APL who either presented with ICB or developed one during induction therapy from January 1998 to May 2022. A total of 488 patients were treated during the study period (Fig. 1). Of these, we excluded 37 cases (3 were treated at relapse, 8 had received partial treatment at another center, 21 were discharged against medical advice without evidence of ICB during induction, and 5 were on induction therapy at the time of this analysis). Of the remaining 451 patients who were treated for newly diagnosed APL at our center, 280 were treated with single-agent ATO and minimal anthracyclines (treated prior to the year 2015) [2], and 171 were treated with ATO, all-trans retinoic acid and minimal anthracyclines (after the year 2015) [3]. Of these, 32 (7.1%) patients had presented with an ICB while another 14 (3.1%) patients developed an ICB during induction therapy. Their characteristics (*n* = 46; 10%) are as shown and compared with the rest of the cohort without an ICB (*n* = 405; 90%) in Table 1. Patients with ICB had lower hemoglobin, higher WBC counts, lower platelet count, higher LDH, and higher blasts + promyelocytes in blood and marrow as compared to those without intracranial bleed. Of the 32 patients who presented with ICB, 13 (40.6%) died during induction while one patient was discharged against medical advice and the remaining 18 survived and were in morphologic remission at the end of induction. Of the 14 patients who developed ICB during induction therapy at a median of day 4 (IQR: 4 to 15 days), 10 (71.4%) died during induction therapy, 1 was discharged against medical advice while the remaining 3 survived and were in morphologic remission at the end of induction. Of the 405 patients who did not have ICB at presentation or during induction, 32 (7.9%) died during induction while 1 patient did not achieve CR post-induction. The remaining 372 patients achieved morphologic remission at the end of induction. A total of 21 of 46 patients (45.7%), who had ICB either at presentation or during induction, were in morphologic remission at the end of induction. This group (n = 21) had a similar event-free survival (event as death or relapse) as compared to patients who did not have ICB and were in remission following induction (*n* = 372) (5-year EFS 88.9% ± 7.4% vs 77.8% ± 2.4%, log-rank p 0.683) (Supplementary Fig. 1). The median follow-up time for this cohort was 53 months (IQR: 27 to 101). Treatment with single-agent ATO ± anthracycline was associated with worse EFS on multivariate analysis in this cohort (Supplementary Table 1). Even, on stratifying patients based on the treatment received (single agent ATO ± anthracycline and ATO + ATRA ± anthracycline), patients who had ICB and were in remission after induction had similar event-free survival as compared to patients who did not have ICB and were in remission following induction (Supplementary Fig. 2). In patients who were treated with ATO + ATRA ± anthracycline, the 3-year EFS for patients with ICB who were in remission after induction was 100% versus 95.4% ± 2.3% in those who did not have ICB and were in remission after induction.

**Table 1:** Comparison of the characteristics of patients with APL who presented with or developed intracranial bleeding during induction therapy (*N* = 46) versus those who did not have intracranial bleeding (*N* = 405) (Laboratory parameters are from the time of diagnosis of APL).

Variables	Patients with intracranial bleed (*N* = 46)	Patients without intracranial bleed (*N* = 405)	*P* value
	Mean ± SD/Median (IQR)/N(%)	Mean ± SD/Median (IQR)/*N*(%)	
Age	28.5 (22,37)	32 (23,43)	0.177
Male gender	26 (56.5)	221 (54.6)	0.801
Duration of symptoms in days	14 (7,28)	14 (7,28)	0.321
Proportion with WBC counts at presentation above 10 × 10^**9**^/L	28 (60.8)	160 (39.5)	0.055
Hemoglobin at presentation (g/dL)	7.1 ± 2.4	8.1 ± 2.5	**0.007**
WBC counts at presentation (×10^**9**^/L)	44.2 ± 51.1	19.9 ± 33	**<0.001**
Platelets at presentation (×10^**9**^/L)	14.8 ± 11.9	28.9 ± 34.5	**<0.001**
Blasts+promyelocytes in blood(%)	79.4 ± 21.2	50.2 ± 35.3	**<0.001**
Prothrombin time (in s) at presentation	15.8 ± 3.3	14.8 ± 5	0.177
Activated partial thromboplastin time (in s) at presentation	30.5 ± 7.5	30.2 ± 6.2	0.793
Creatinine at presentation (in mg/dL)	0.87 ± 0.18	0.87 ± 0.32	0.931
Fibrinogen (in mg/dL)	197.7 ± 123.2	217.5 ± 134.2	0.325
Bone marrow blasts+promyelocytes (%)	88 ± 10	80 ± 15	**<0.001**
LDH at presentation (in IU/L)	1177 ± 742	791 ± 653	**<0.001**
Differentiation syndrome	4 (8.7)	42 (10.4)	0.477
Induction mortality	24 (52.2)	32 (7.9)	**<0.001**

The median age of both cohorts (ICB and those without ICB) was lower than typical cohorts from the US and European countries, most likely due to a referral bias of tertiary care data combined with the younger population structure in developing countries [4]. The median time from the onset of symptoms to diagnosis was 2 weeks. This delay in diagnosis possibly could have contributed to the higher rate of ICB at presentation within the ICB cohort.

From this retrospective analysis, we conclude that while using an upfront ATO-based regimen, intracranial bleeding at presentation or during induction does not seem to be associated with an increased risk of relapse of APL.

## Supplementary information

Supplementary

## Data Availability

The datasets generated during and/or analyzed during the current study are available from the corresponding author upon reasonable request.
