# Supplementary material for: Intracranial bleeding in acute promyelocytic leukemia treated with arsenic trioxide based regimens is associated with induction mortality but not with relapse
Source: Blood Cancer J. 2023 Jun 22;13(1):94. doi: 10.1038/s41408-023-00873-z (PMC10287743; doi:10.1038/s41408-023-00873-z)

**Supplementary Table 1: Multivariable Cox Proportional hazard model for event free survival for patients with APL surviving induction therapy (N= 393)**

| **Variable** | **HR (95% - CI)** | **p value** |
| --- | --- | --- |
| **Age (in years)** | 0.98(0.96 – 0.99) | **0.015** |
| **Male gender** | 1.86(1.18 – 2.93) | **0.007** |
| **Total WBC counts: >10 x 10^9^/L*** | 1.40(0.90 – 2.18) | 0.134 |
| **Group*** |  |  |
| IC bleed | 1.08 (0.34 – 3.49) | 0.895 |
| No IC bleed | 1.00 |  |
| **Type of treatment received** |  |  |
| ATO + ATRA ± anthracycline | 1.00 | **0.001** |
| Single agent ATO ± anthracycline | 4.42(1.89 – 10.31) |  |

* Statistically significant and clinically important variables were included in the multivariable analysis.

**Supplementary Figure 1**: Event free survival of patients with APL with IC bleed who survived induction therapy and were in morphologic remission at the end of induction (N=21) versus those who did not have intracranial bleeding and were in morphologic remission at the end of induction (N=372) (5 year EFS 88.9% ± 7.4% v/s 77.8% ± 2.4%, log rank p 0.683).


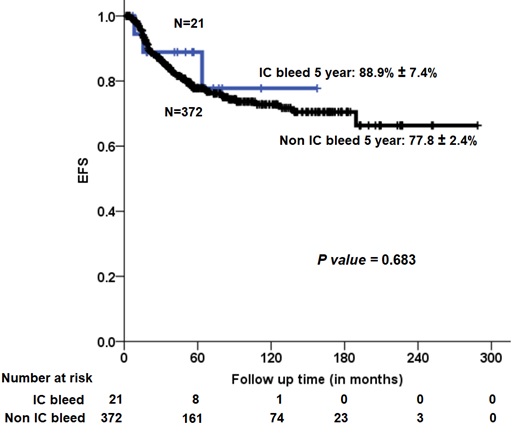


**Supplementary Figure 2: A.** Event free survival of patients with APL with IC bleed, treated with single agent ATO ± anthracyclines, survived induction therapy and were in morphologic remission at the end of induction (N=8) versus those who did not have IC bleed, treated with single agent ATO ± anthracyclines, survived induction therapy and were in morphologic remission at the end of induction (N=258) (5 year EFS 71.4% ± 1.7% v/s 72.9% ± 2.9%, log rank p 0.435). Median follow up time in months: 74 (IQR: 34 to 129). **B.** Event free survival of patients with APL with IC bleed, treated with ATO + ATRA ± anthracyclines, survived induction therapy and were in morphologic remission at the end of induction (N=13) versus those who did not have IC bleed, treated with ATO + ATRA ± anthracyclines, survived induction therapy and were in morphologic remission at the end of induction (N=114) (3 year EFS 100% v/s 95.4% ± 2.3%, log rank p 0.336) Median follow up time in months: 37 (IQR: 20 to 54).


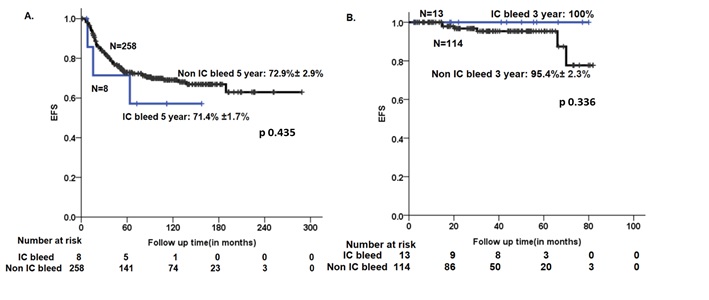

Supplement: Supplementary file 1 — Supplementary [file 41408_2023_873_MOESM1_ESM.docx]
